# Gene Expression Variability within and between Human Populations and Implications toward Disease Susceptibility

**DOI:** 10.1371/journal.pcbi.1000910

**Published:** 2010-08-26

**Authors:** Jingjing Li, Yu Liu, TaeHyung Kim, Renqiang Min, Zhaolei Zhang

**Affiliations:** 1 Department of Molecular Genetics, University of Toronto, Toronto, Canada; 2 Donnelly Centre for Cellular and Biomolecular Research, University of Toronto, Toronto, Canada; 3 Banting and Best Department of Medical Research, University of Toronto, Toronto, Canada; 4 Department of Computer Science, University of Toronto, Toronto, Canada; Tufts University, United States of America

## Abstract

Variations in gene expression level might lead to phenotypic diversity across individuals or populations. Although many human genes are found to have differential mRNA levels between populations, the extent of gene expression that could vary within and between populations largely remains elusive. To investigate the dynamic range of gene expression, we analyzed the expression variability of ∼18, 000 human genes across individuals within HapMap populations. Although ∼20% of human genes show differentiated mRNA levels between populations, our results show that expression variability of most human genes in one population is not significantly deviant from another population, except for a small fraction that do show substantially higher expression variability in a particular population. By associating expression variability with sequence polymorphism, intriguingly, we found SNPs in the untranslated regions (5′ and 3′UTRs) of these variable genes show consistently elevated population heterozygosity. We performed differential expression analysis on a genome-wide scale, and found substantially reduced expression variability for a large number of genes, prohibiting them from being differentially expressed between populations. Functional analysis revealed that genes with the greatest within-population expression variability are significantly enriched for chemokine signaling in HIV-1 infection, and for HIV-interacting proteins that control viral entry, replication, and propagation. This observation combined with the finding that known human HIV host factors show substantially elevated expression variability, collectively suggest that gene expression variability might explain differential HIV susceptibility across individuals.

## Author Summary

Many human genes have population-specific expression levels, which are linked to population-specific polymorphisms and copy-number variations. However, it is unclear whether human genes show similar dynamic range of expression between populations. In this work we analyzed HapMap gene expression compendium, and quantified the between-population and within-population expression variability for ∼18,000 human transcripts. We first concluded that the majority of the human genes have similar levels of within-population variability. However, a small fraction (∼4%) does show much higher expression variability in one population, and the deviation is consistently associated with increased SNP heterozygosity in their UTR regulatory regions. We further showed that genes with the greatest within-population expression variability are significantly enriched for chemokine signaling associated with HIV-1 infection. Combined with the finding that human HIV-1 host factors tend to have increased expression variability within populations, our analysis may explain, at least in part, different susceptibility to HIV infection within the human population. This work provides a fresh angle for analyzing gene expression variations in populations.

## Introduction

In both prokaryotic and eukaryotic organisms, variations in gene expression exist widely within and between populations, which can be attributed to either genetic or non-genetic factors. Genetic factors are changes in DNA sequence that cause expression differences, such as single nucleotide polymorphisms (SNPs) and copy number variations (CNVs) on expression qualitative trait loci (eQTLs) [1], [2]. Non-genetic factors include epigenetic modifications [3], [4] and also innate expression stochasticity at the single-cell level [5], [6]. To date, extensive studies have investigated gene expression variation within and between natural populations of yeast [7], [8], fly [9]–[11], fish [12]–[14] and human [1], [2], [15]–[17]. These studies were mostly focused on identifying genes showing differential expression between populations or on localizing causal elements that affect expression changes among individuals (eQTL mapping). However, expression variation, as a manifested phenotype, in and of itself has complicated functional implications. It is established that the onset of many human diseases was associated with expression variation of some crucial genes [18], [19], and therefore gene expression variation is likely to be subject to selection. In this sense a systematic study on the *expression variability* within human populations is needed, which delineates the dynamic range of gene expression, *i.e.* to what degree a gene's expression could vary across individuals. This is of particular importance for several reasons. First, *expression variability* is conceptually distinct from *differential expression* (difference in *mean expression level* between populations); therefore studying expression variability might shed light on the evolution and differentiation of human gene expression. In analogy to sequence evolution, if a new advantageous expression level is rapidly fixed by natural selection in one population, a substantial reduction in expression variability might be expected. Second, expression variability is a natural estimate of dosage sensitivity of human genes. Due to natural selection, expression variability of dosage-sensitive genes is expected to be minimized; therefore investigation of expression variability might pave the way to future study of dosage sensitivity for human genes. Finally, recent genome-wide association studies have been based on the hypothesis of common disease-common variant (often abbreviated CD-CV), which carries the assumption that common variants might cause common aberrant expression of disease-associated genes, giving rise to pathological phenotypes. Given the widespread differential susceptibility to diseases within human populations, by circumventing the identification of causal sequence variants, a direct examination of expression variability of human genes and its implication towards disease susceptibility would highlight the importance of associating expression polymorphism to human disease.

In this paper, we sought to tackle the above questions by investigating the expression variability of human genes based on the previously published whole-genome expression profiling data [1], [2]. We found that, for most human genes, their *within-population* variability does not significantly differ between populations, with only a small group of genes exhibiting population-specific expression variability. Furthermore, this set of variable genes has SNPs in their untranslated regions (both 5′ UTRs and 3′UTRs) that show a pronounced elevated difference in population heterozygosity, which might explain, at least partially, their deviant expression variability between populations. We also found that a majority of human genes shows substantially reduced within-population variability, prohibiting the genes from differential expression between populations. Functional enrichment analysis revealed that genes with higher within-population variation are involved in a number of human diseases, particularly the early stage of HIV-1 entry into target cells, suggesting that expression variability is linked to variation in susceptibility to HIV infection among individuals.

## Results

### The expression variability of most human genes is consistent between populations

The recently released whole-genome expression profiling data include 270 HapMap individuals spanning 4 ethnic populations [1], [2], including CHB (Chinese Han in Beijing), YRI (Yoruba people of Ibadan, Nigeria), CEU (U.S. residents with northern and western European ancestry) and JPT (Japanese from Tokyo). After preprocessing the expression data, we compiled expression profiles of 18, 081 human mRNA transcripts across all HapMap populations (CEU/YRI unrelated children, CEU/YRI unrelated parents, CHB and JPT, see **Materials and Methods**). After filtering out the Y-linked genes, we included both male and female samples since sex-biased expression is minimal (even for X-linked genes) in the lymphoblastoid cell line [20]. Although the subsequent analysis was based on CEU and YRI adult children (30 individuals in each population), all the conclusions hold for CEU/YRI parents, and also CHB and JPT, unless otherwise mentioned (see **Figures S1, S2, S3, S4, S5**).

**Figure 1:**
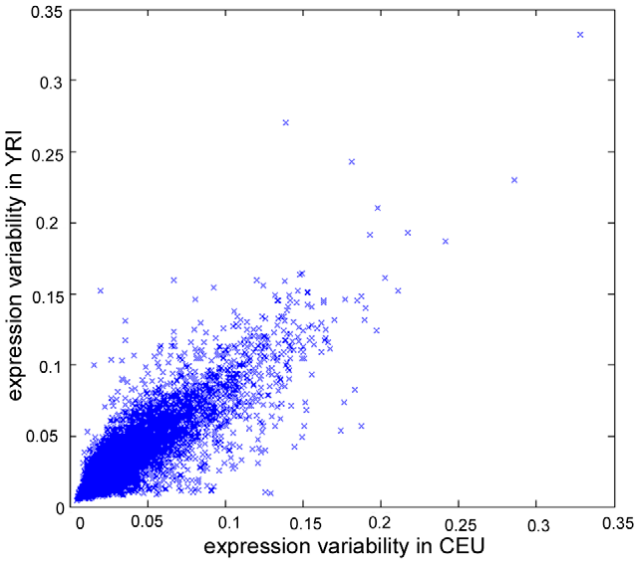
Correlation of expression variability between CEU and YRI populations. Each data point represents one transcript.

We first sought to examine whether these genes have similar level of within-population variability in different populations. For each gene, we quantified the within-population expression variability by calculating its coefficient of variation η, which is the ratio of the standard deviation of its expression (across 30 individuals within a population) to the mean value [21]–[23]. Although other metrics can be used to quantify the expression variability, η is known to be one of the most robust and unbiased metrics [21]. Greater η implies higher expression variability for a particular gene across individuals within a population, while a significant reduction in η suggests that the gene might be dosage sensitive and thus under severe selection to minimize expression variability. The η values were calculated for each of the 18,081 mRNAs across individuals within the CEU and YRI populations separately (see **Table S1** for genes with their calculated expression variability in each population). Between the CEU and YRI populations, most of the human genes exhibit a similar level of within-population variability, as η in CEU is well correlated with that in YRI (r = 0.88, P≈0; Figure 1). Pair-wise comparison of expression variability between all HapMap populations further confirmed this trend (r>0.85, P≈0). The same trend was recapitulated on another independent dataset of smaller sample size based on Affymetrix Human Focus Arrays [16], suggesting this observation was not resultant from a technical artifact. Therefore such a strong correlation of within-population expression variability between the two populations suggests either expression variability of most genes is subject to similar levels of constraints in both populations, or the *cis-* or *trans-* regulatory mechanisms of these genes have not diverged significantly.

Although the within-population variabilities of most human genes are tightly correlated between populations, a small number of genes do show noticeably different level of variability between CEU and YRI (Figure 1). To systematically identify those outliers with population-specific expression variability, we reciprocally regressed the values of η based on a linear model with random effects. Using residual analysis (see **Materials and Methods**) we were able to identify 919 and 898 genes as outliers for η's in YRI and CEU respectively as the explanatory variables. Among these outlier genes, 711 were found to be independent of the direction of the regression (either regressing η_CEU_ with η_YRI_ or regressing η_YRI_ with η_CEU_, see **Table S2** for a complete gene list). We noticed the presence of some annotated SNPs on the Illumina probes (affecting 4.5% of the 711 variable genes), so we removed the affected genes and only considered the remaining 679 outlier genes in our following analysis. We also noted that, among all the human genes, about 5% (916/18,081) had a probe overlapping with SNPs; this percentage is statistically indistinguishable from the percentage for the outlier genes (5% vs 4.5%, P-value = 0.50, Chi-square test). We thus eliminated the possibility that the observed expression variability was caused by the existence of SNPs in the microarray probes.

### *Cis*-SNPs on UTRs of variable genes show elevated difference in population heterozygosity

Could the observed asymmetric expression variability between populations be explained by their associated sequence variants? Supposing expression of a gene is only affected by a causative bi-allelic SNP, it is expected that the SNP with similar minor allele frequencies (MAFs) in both populations should have comparable expression variability of the associated gene. In other words, the observed increased expression variability of a particular gene is likely to be associated with some causative SNPs with divergent MAFs between two populations. Particularly under the assumption of Hardy-Weinberg Equilibrium for the diploid human populations, MAF of a SNP can be used to infer its expected heterozygosity *θ* (fraction of the heterozygous genotype) within a population [24]. Thus if a gene shows elevated expression variability in one population, the sequence variants affecting this gene are likely to have elevated expected heterozygosity within the population. Due to the difficulties in identifying *trans*-acting factors, we set out to examine this possibility for *cis-*SNPs surrounding the 679 genes showing population-specific expression variability.

**Figure 2:**
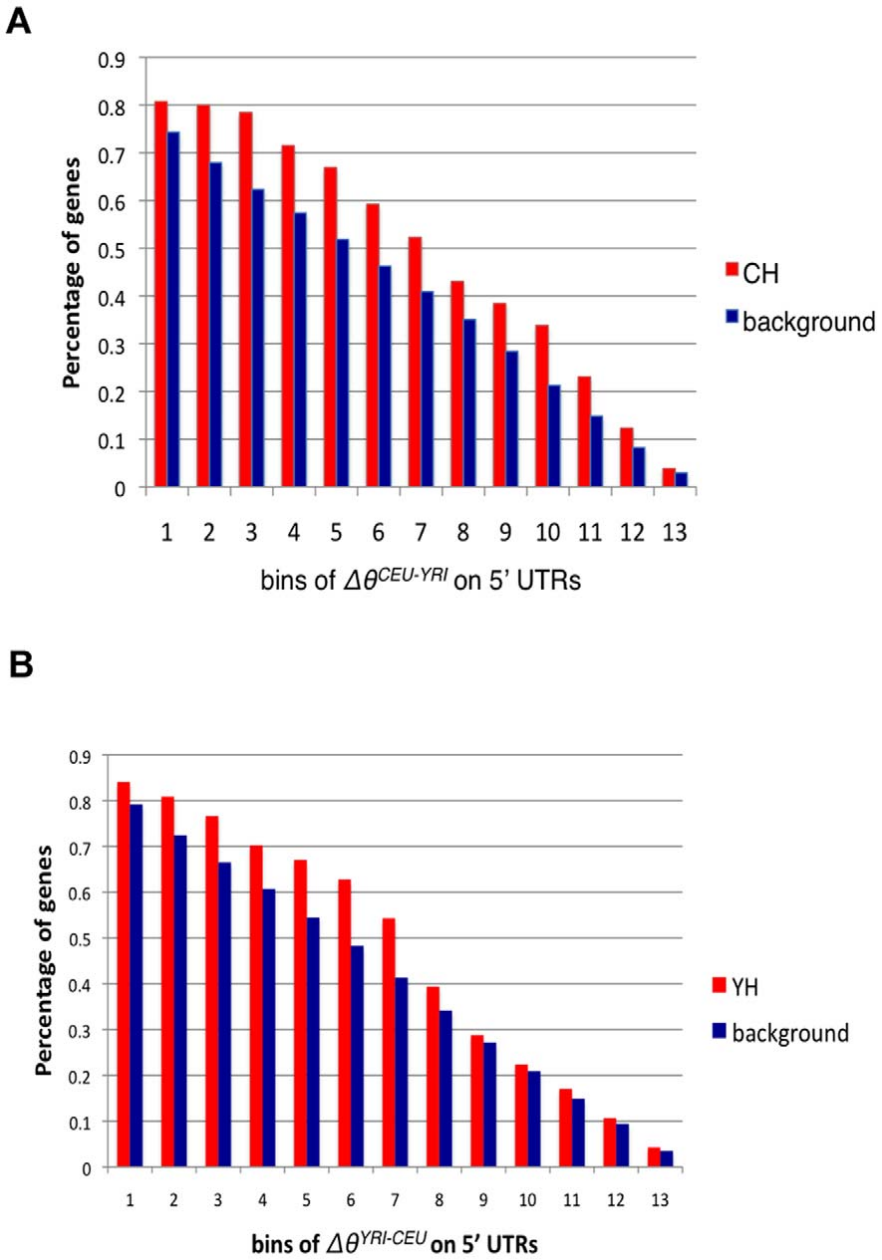
Genes that have higher within-population expression variation have higher expected heterozygosity than genome background. (**A**) Comparison of expected heterozygosity between background genes and CH genes. For each group we calculated the percentage of genes having at least a SNP in its 5′UTR with *Δθ^CEU-YRI^* greater than a given cutoff, *k*, varying from 0 to 0.5 (the maximal *Δθ^CEU-YRI^*) with an increment of 0.04, which gives 13 bins. (**B**) Comparison of expected heterozygosity between background genes and YH genes. For each group we calculated the percentage of genes having at least a SNP in its 5′UTR with *Δθ^YRI-CEU^* greater than a given cutoff, *k*, varying from 0 to 0.5 (the maximal *Δθ^YRI-CEU^*) with an increment of 0.04, which gives 13 bins.

We downloaded the promoter, 5′ UTR and 3′ UTR sequences for all human RefSeq genes (>20, 000) from UCSC Genome Browser [25], and mapped ∼3 million HapMap Phase II SNPs onto them (see **Materials and Methods**). We first examined the SNPs on 5′UTRs. We divided the 679 most variable genes into two groups: genes showing higher expression variability in CEU (383/679, termed CH group), and the remaining genes (296/679) showing higher expression variability in YRI (termed YH group). With the current SNP annotation, we were able to map SNPs onto the 5′ UTRs of 5, 690 human genes, including 130 CH genes and 94 YH genes. For each SNP on the CH genes, we calculated its difference in expected population heterozygosity between CEU and YRI (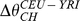), and the same calculation was performed for all the SNPs on all the mapped 5, 690 human genes as background control (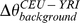). As CH genes show elevated expression variability in CEU than in YRI, by comparing with genome background, we next tested if they are enriched for genes associated with higher population heterozygosity in CEU than in YRI (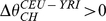). As each gene often has multiple SNPs on its 5′UTRs, we first selected a cutoff, *k*, varying from 0 to 0.5 (the maximal 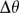) with an increment of 0.04, and then compared the percentage of genes in each group (CH genes and background genes) bearing at least one SNP with 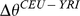 greater than this cutoff. As seen in **Figure 2(A)**, for all the cutoffs used, the CH genes consistently showed higher percentage than the genome background. To determine the statistical significance, we chose to use a stringent cutoff *k* = 0.04 (instead of using *k* = 0 to avoid numerical fluctuation), and found the percentage of genes in CH group bearing at least one SNP with 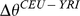
>*k* is significantly higher than the genome background (P = 3.4×10^−3^, χ^2^ test). Similarly for YH genes, population heterozygosity was compared between YRI and CEU; thus 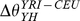 and 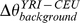 were calculated for each YH SNPs. With the same analysis, as shown in **Figure 2(B)**, we reached the same conclusion that YH genes are significantly enriched for genes with elevated population heterozygosity in YRI (P = 0.05, χ^2^ test).

For 3′ UTR SNPs, we found the same enrichment for CH genes (P = 1.5×10^−3^, χ^2^ test), but not for the YH genes (P = 0.8, χ^2^ test). Moreover, neither CH nor YH genes show the trend on promoter SNPs (P>0.3, χ^2^ test). Taken together, the observed unequal expression variability between populations is likely to be explained, at least in part, by uneven MAF and population heterozygosity of the SNPs on UTR regions.

**Figure 3:**
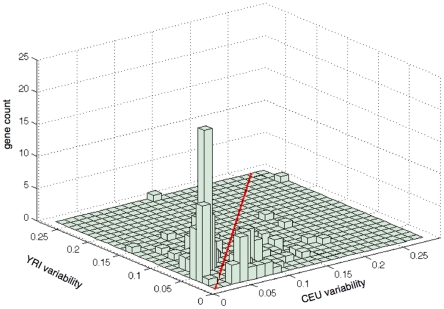
The distribution of expression variability for the 184 differentially expressed genes. The red diagonal line on the horizontal plane is a reference line, indicating equal expression variability in both populations.

Among the 679 outlier genes that showed population-specific expression variability (see above), we were able to identify 184 genes that have differentiated expression levels between CEU and YRI (FDR≤0.01, 10,000 random permutations) after Benjamini and Hochberg FDR correction (see **Materials and Methods**), *i.e.* these genes on average have significantly higher expression levels in one population than in the other. For each of these 184 transcripts, we then plotted the distribution of within-population expression variabilities in CEU and YRI as a histogram in **Figure 3**, where the red diagonal line on the horizontal plane indicates equal expression variability in both CEU and YRI. Strikingly, we found among the total 184 transcripts, far more genes had higher expression variability in YRI (105 genes, 57%) than in CEU (79 genes or 43%). As we described in the above sections, among the total 679 outlier genes, 44% had higher expression variability in YRI, while among the 184 differentially expressed genes, a subset of the 679 outlier genes, the percentage substantially increased to 57%. With 10, 000 random permutation test, we confirmed such an enrichment of genes with higher expression variability in YRI is highly significant (P<10^−5^). Although the conclusion was drawn from 30 unrelated adult children from CEU and YRI, it also holds for the 60 unrelated parents in the two populations, suggesting our results are robust against sample size.

### Reduced gene expression variability between populations

**Figure 4:**
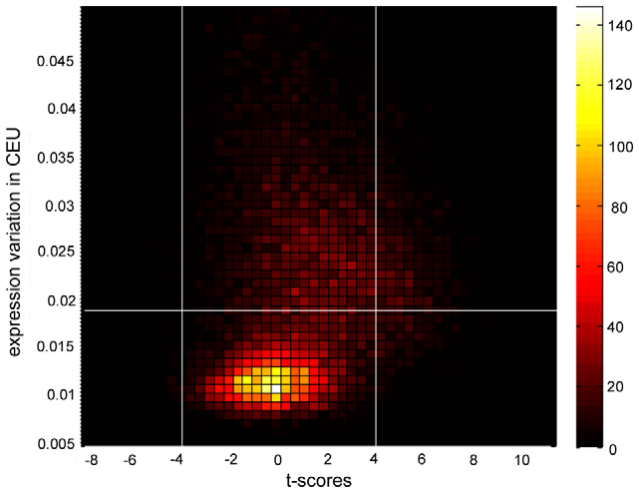
2D histogram of t-scores between CEU and YRI and expression variation in CEU. Only genes with variability smaller than 0.05 (15, 932 out of 16,878) are presented here. The two vertical lines are thresholds defining differential expression (up- and down- regulation) and the horizontal line indicates expression variability of genome background, which is the median across all the surveyed transcripts.

Among the majority of genes that have similar within-population expression variability in CEU and YRI (the non-outlier genes, see **Materials and Methods**), we also detected ∼20% (3, 429) that show differential expression levels between these populations with FDR = 0.01 (Benjamini and Hochberg FDR correction). Combined with the fact that only 184 among the 679 outlier genes (27%) show differential expression levels (see the above section), this clearly suggests the divergence of gene expression between populations is mostly manifested as a significant shift in expression levels without affecting within-population variability. We further quantified the degree of differential expression for each transcript between CEU and YRI through t-scores derived from a standard t-test (see **Materials and Methods**), which is the standardized distance of mean expression level between two populations. Higher absolute value of t-score is equivalent to a lower p-value, e.g. t = ±2 corresponding to p = 0.05 before Bonferroni correction, and t = ±5 corresponding to p = 0.05 after Bonferroni correction. As expression variability between CEU and YRI is almost perfectly correlated after removing the outliers in this study (r = 0.94), we only compared t-scores and expression variability for the transcripts in CEU (**Figure 4**, in which we used t = ±4 as a threshold to define differential expression levels between the populations, indicated by the two vertical lines, approximately corresponding to p = 2×10^−4^). As shown in **Figure 4**, a majority of genes has t-scores centered on 0 and has substantially reduced within-population expression variability compared with the genome background (the horizontal line). This observation indicates that a significant reeducation in expression variability within a population prohibits the genes from differential expression between populations. This group of genes is likely to be dosage-sensitive, which requires them to have similar expression levels between populations. It is also clear from **Figure 4** that some genes have similar expression levels between two populations but also have very high expression variability (above the horizontal line); this implies these genes might be more dosage tolerant. We further noted a significant positive correlation between t-score and expression variability (r = 0.18, P<0.01) for genes shown in **Figure 4**, suggesting that genes with higher expression variability are more likely to develop more divergent expression levels between populations. Thus high expression variability is likely to confer higher expression evolvability. The conclusion stands when using another approach to identify the differentially expressed genes, which considers potential batch effects at the establishment of the cell lines [2].

### Genes with the highest within-population expression variability are linked to disease susceptibility

Next we sought to determine whether genes with extreme within-population variability are specifically involved in any maladaptive processes. Since we are now studying the global trend of expression variability of human genes, we sought to exclude the genes that have population-specific expression variabilities. We excluded 1,106 of such genes from the total list of 18, 081 genes by either regressing η_CEU_ with η_YRI_ or regressing η_YRI_ with η_CEU_ (the union set, compared with the outliers as intersection set described above). In the end we retained a total of 16,975 mRNAs that showed similar variability in CEU and YRI. Since these transcripts have highly correlated within-population variability between these two populations, we focused the following analysis only on CEU population, unless otherwise mentioned.

The 16,975 mRNAs with homogeneous variability in the two populations were ranked according to their expression variability η from the lowest to the highest. By controlling the confidence level at 5%, we selected the top 2.5% and bottom 2.5% as the most and the least variable genes for further comparison respectively (424 out of 16, 975 genes for each group, see **Table S3** for complete lists of genes). We performed an enrichment test by setting all 16, 975 transcripts in our study as background, then applied subsequent false discover rate (FDR) correction on each functional category using classifications in the DAVID biological database [26]. Functional enrichment analysis specifically included (1) Gene Ontology (GO) classifications (biological process, cellular component and molecular function at all levels), (2) KEGG pathways, (3) interaction with HIV-1 (human immunodeficiency virus 1) (from *NCBI HIV-1*, *Human Interaction Database* [27]), and (4) human disease annotations (from NIH Genetic Association Database [28] and OMIM).

As shown in Table 1, genes with the lowest expression variability are significantly enriched for fundamental biological processes such as *translation* and *ribosome constituents* (FDR = 0.02). The ribosomal genes are known to be dosage-sensitive [29]; this observation strongly suggests that expression variability within human populations indeed reflects intrinsic dosage-sensitivity of human genes. In sharp contrast with the least variable genes, genes with the greatest variability are enriched for *behavior* (FDR = 0.08), *taxis* (FDR = 0.02) and *response to external stimulus* (FDR≤0.05). While genes with the least expression variability are not associated with any human diseases reported from case-control studies deposited in GAD (NIH Genetic Association Database [28]), interestingly, genes with the highest expression variability are associated with seven human diseases (Table 1), mostly related to disease classes including ageing (FDR = 0.007) and neurological disorders (FDR = 0.036). Examination using disease associations documented in OMIM (Online Mendelian Inheritance in Man) did not find significant associations, however this might be due to the lower coverage of OMIM as compared to GAD, and the more stringent criteria used by OMIM in reporting disease associations. As GAD is primarily designed for collecting disease-associated genes bearing unevenly distributed biomarkers (e.g. SNPs), our observed disease association might be attributed to expression manifestation of these documented sequence polymorphisms.

**Table 1:** Enriched functional categories for genes showing the least and the most expression fluctuation (FDR<0.1).

	GO-Biological Process	GO-Cellular Component	GO-Molecular Function	Disease Association
Genes showing the **least** expression variability	neuropeptide signaling pathway	cytosolic ribosome	constituent of ribosome	
	neurological system process	ribosomal subunit	transmembrane receptor activity	
	translation	large ribosomal subunit	rhodopsin-like receptor activity	
	multicellular organismal process	small ribosomal subunit	molecular transducer activity	
	cell surface receptor linked signal transduction	plasma membrane		
Genes showing the **most** expression variability	anatomical structure morphogenesis	plasma membrane	integrin binding	hepatocellular carcinoma
	chemotaxis	integral to Golgi membrane	receptor binding	osteoarthritis
	immune response		cytoskeletal protein binding	psoriasis
	cell morphogenesis		chemokine activity	heart disease, ischemic
	behavior		chemokine receptor binding	asthma
	locomotory behavior		cytokine binding	skin cancer, non-melanoma
	cell communication			Q fever
	cytosolic calcium ion homeostasis			
	response to external stimulus			**Associated Disease Class**
				aging
				neurological

### Gene expression variability and HIV infection

In addition to being enriched for disease annotations listed above, genes with the highest expression variability also show significant enrichment for interaction with two HIV-1 proteins (see **Materials and Methods**). Notably, the highly variable genes are associated more frequently with the HIV-1 gene *env* (the precursor to HIV surface glycoprotein gp120; FDR = 0.018), and preferentially up-regulate the other HIV-1 gene, *tat* (FDR = 0.0024), whose protein product is of vital importance in regulating viral replication. Worthy of note, the HapMap samples used in this study were derived from lymphoblastoid B cells while the natural targets of HIV-1 are CD4^+^ T cells; however recent *in vitro* experiments have established that the lymphoblastoid cell line derived from B cells can well reflect the behavior of CD4^+^ T cells upon the infection of HIV-1 [30]. Therefore our observations suggest that the variation among individuals in their susceptibility to HIV viral entry or replication might be linked to the elevated expression variability of the host genes interacting with *env* and *tat*. Further lending support to this hypothesis, we found that variable genes are also enriched for chemokine receptors (FDR = 0.08). Since the HIV-1 virus fuses into target cells mainly through interactions between gp120 and chemokine receptors (e.g. CXCR4 and CCR3), this strongly supports that variability across populations is inherently linked to varied susceptibility to HIV-1.

**Figure 5:**
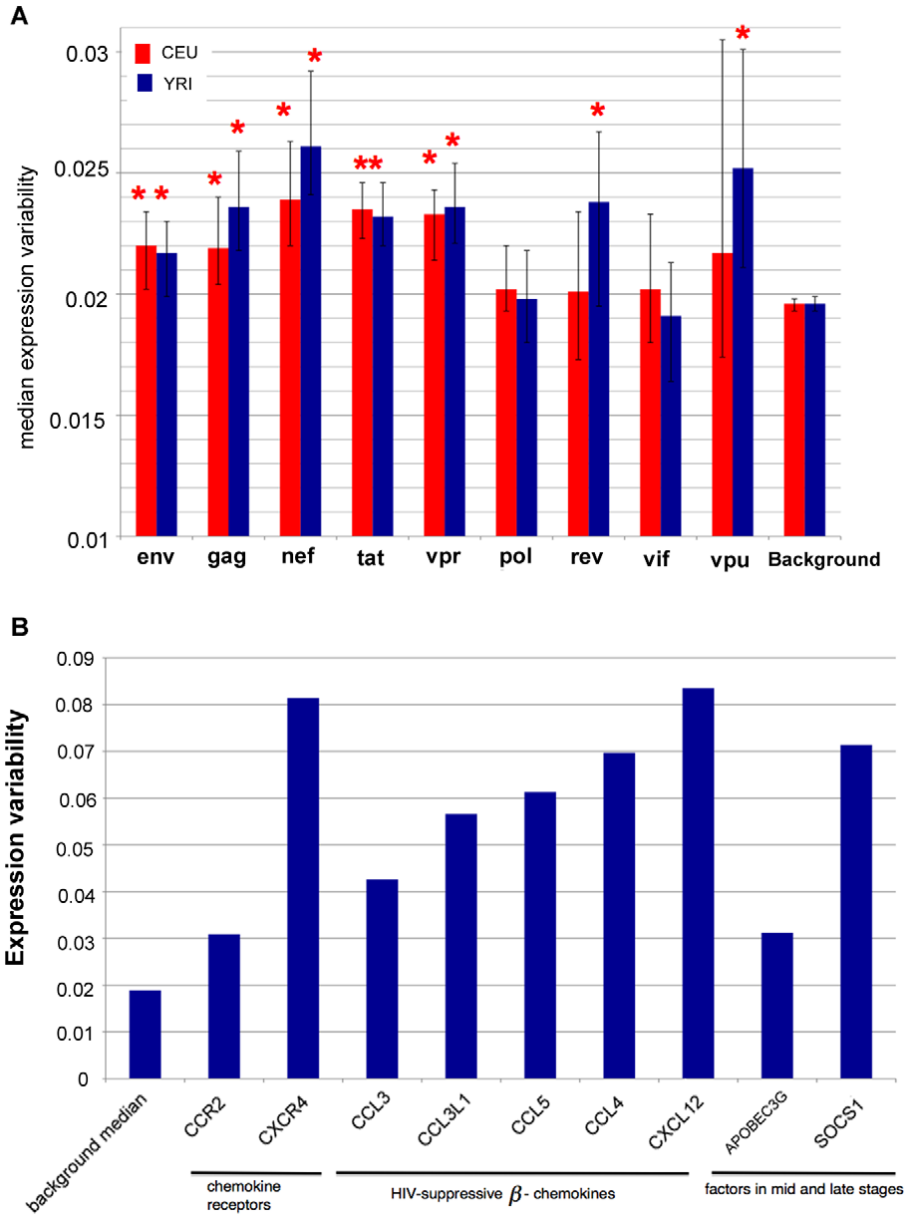
Human genes involved in HIV infection have higher expression variability. (**A**) Human genes interacting with HIV proteins show elevated expression variability. P-values less than 0.05 are indicated by asterisks. Error bars are 95% confidence intervals derived from 5, 000 bootstrap re-sampling. (**B**) Key factors affecting HIV susceptibility in literature show elevated expression variability compared with that of the genome background.

The HIV-1 genome consists of 9 genes: *env*, *gag*, *nef*, *pol*, *rev*, *tat*, *vif*, *vpr* and *vpu*. To further explore the strong association between expression variability of host genes and HIV-1 pathogenesis, we next compared the expression variability of human host factors interacting with each of the 9 viral genes against human genome background (for CEU and YRI separately). The host-virus interactions were extracted from *HIV-1*, *Human Protein Interaction Database* [27]. We were able to identify 700, 194, 235, 211, 73, 853, 83, 215 and 30 human transcripts in our data set that have annotated interactions with the 9 HIV-1 genes respectively, and we examined the interactions in all categories (e.g. physical interaction, up-regulate or down-regulate, etc.). Strikingly, for 5 of the 9 HIV-1 genes (*env*, *gag*, *nef*, *tat* and *vpr*), the host factors exhibited significantly elevated expression variability in both populations (all p-values<0.05, Wilcoxon ranksum test; **Figure 5a**). For *rev* (regulator of virion) and *vpu* (viral protein U), only YRI population exhibited elevated expression variability (note that the relatively large error bars for *vpu* in both populations were due to small sample size as only 30 human genes were annotated to interact with *vpu*).

As the genome-wide expression profiling was performed in the lymphoblastoid cell line (an immune-related cell line that HIV virus can attack), combined with the observation that genes involved in immune system are enriched among the host factors interacting with the viral genes (P-value<0.05), it is tempting to trivially explain the above observation by the intrinsic variability of immunity genes [31]–[33]. To ascertain this possibility, we identified 361 human transcripts (∼16% of all the host factors in this study) that contain the keyword “immune” in their Gene Ontology annotations (Biological Processes, all hierarchies), and removed them from the host factors and repeated the above comparison. Again, we found host factors interacting with *nef* (negative regulatory factor), *tat* (trans-activator of transcription) and *vpr* (viral protein R) constantly show elevated expression variability in both CEU and YRI, which suggests that the elevated expression variability of the host genes cannot be fully explained by the enrichment of the immunity genes.

After ruling out the effect of immunity genes, we next applied two approaches to ascertain the possibility that the elevation of expression variability for HIV-interacting genes could be due to enrichment of highly variable GO functional categories. (i) Firstly, we pooled together the entire 1, 480 human genes that were annotated to interact with at least one HIV-1 genes, and removed 551 genes associated with the highly variable functions (based on GO terms derived from Table 1 and Supplemental Table S4, we removed all genes associated with these GO terms and their descendents in the GO hierarchy). For the remaining 929 HIV-interacting genes, again we observed their within-population expression variability is significantly higher than genome background in both CEU and YRI (showing ∼17% increase in comparison with expression variability of all human genes, P<10^−11^, Wilcoxon ranksum test). (ii) In the second approach, we generated “null” sets of genes, mirroring the GO functional categories of the 1,480 HIV-1 interacting genes and compared the variability of these null sets to the real gene set. Among the 1, 480 genes, we were able to consider 1, 284 genes, whose GO annotations (the most specific code) were also associated with at least one non-HIV interacting gene. We then chose a non-HIV-interacting gene with the same GO code and repeated this for every one of the 1,284 genes to make a null set. We repeated this procedure 1000 times by generating 1000 null gene sets, and asked among the 1000 simulations, how many times we observe the real data have significantly higher expression variability than the null set. Consistently, we found in all simulations, the real data always have average higher expression variability (on average 8% higher), and 991 out of the 1000 simulations are statistically significant. Thus we concluded that the observed elevation in expression variability of HIV-interacting genes is unlikely an artifact caused by the bias in the GO functional annotations.

Next we curated a list of human genes from the published literature that are known to induce differential susceptibility to HIV, and compared their expression variability with the genomic background. These genes included chemokine receptors (CCR2 [34]–[36], CXCR4 [37]), HIV-suppressive β-chemokines (CCL3 [38], CCL3L1 [39], CCL4 [40], CCL5 [41], [42], CXCL12 [43], [44]), a human endogenous HIV-1 replication inhibitor known to be involved in the mid stage of viral propagation (APOBEC3G [45]), and a newly identified inducible host factor implicated in the late stage of HIV-1 replication pathway (SOCS1) [46]. As shown in **Figure 5(b**), these key host factors have substantially elevated expression variability as compared to the genomic background. For example, CXCR4, one of the major chemokine receptors, has an almost 4.3-fold increase in expression variability, suggesting that it might have extremely low expression level in some individuals, leading to increased resistance to HIV entry (particularly for X4 strain, which utilizes CXCR4 for viral entry). Although we did not observe significantly elevated expression variability for CCR5 (η = 0.02, slightly higher than the genome background), we indeed found its ligand CCL3L1 had a 3-fold increase in expression variability. This is consistent with the previous observation that increased copy number of CCL3L1 in some individuals can effectively reduce the risk of HIV-1 infection [39]. Similarly, CXCL12 (SDF-1), the ligand of CXCR4, has a 4.4-fold increase in expression variability. These results collectively bolster the hypothesis that variation in genetic expression within a population may result in altered susceptibility to HIV-1 infection.

We further compared our results with a recent work by Loeuillet *et al* [30], in which the authors established a link between a SNP (rs2572886) to differential HIV susceptibility among European individuals by transduction of lymphoblastoid cells (the same cell line used in our study) with a HIV-1-based vector (HIV.GFP). The identified SNP is associated with 8 genes belonging to the *LY6/uPAR* family, and the authors prioritized 4 proteins (LYP6D, LYPD2, SLURP1 and GML) for over-expression study and 2 proteins (LY6D and LYPD2) for RNAi knockdown. However the authors did not observe HIV infectivity being significantly affected by these perturbations [30]. We re-examined expression variability among CEU individuals for these prioritized proteins, and found their expression variability is substantially below genome average (between 0.009–0.01, compared with the genome median of ∼0.0197). Among the remaining 4 tagged genes that were not examined in the original study, *LY6E* showed almost ∼1.8–2.5-fold increase in expression variability in comparison with that of background genes (expression variability of *LY6E* is 0.049 and 0.035 in CEU and YRI, respectively, in comparison with the background median of ∼0.0197). Therefore a re-examination of *LY6E* might be needed in future studies to elucidate the roles of this gene in affecting HIV susceptibility.

## Discussion

Although extensive efforts have been made to elucidate the effects of sequence variants on expression phenotypes, it is likely that not all expression variation can be fully explained by genetic factors [1], [47]. As gene expression is more pertinent to molecular functions, exploration of expression variability within and between human populations could provide additional insights into functional evolution of human genes. Unlike previous work that had focused on finding genes that are differentially expressed between populations [15], [16], [48], [49] or mapping eQTLs [17], [47], throughout this paper, we quantified expression variability for each human gene within individual human populations, and attempted to interpret the functional and evolutionary implications of such variations.

Our results revealed that the evolution of differential expression in human is largely manifested as a shift in mean expression level between populations without affecting their respective expression variability in each population. As within-population expression variability could be used to approximate dosage-sensitivity of a given gene, our observation also suggests that dosage-sensitivity of human genes is largely conserved between human populations. We also found that differentially expressed genes are more likely to have higher expression variability, which suggests variability might confer higher evolvability due to relaxed constraints.

For those genes that do have significantly different variability between distinct populations (referred as *outliers*), we also observed dissimilar minor allele frequencies (and thus population heterzygosity) between CEU and YRI in their UTRs, particularly on the 5′UTRs. It is possible that in addition to the *cis*-regulatory regions, other trans-acting and non-genetic factors might also take effect.

Our analysis revealed that genes with the highest expression variability within human populations are significantly associated with a number of human diseases, which may account for the differential susceptibility to diseases among human individuals. Although it is expected that sequence polymorphisms tend to be associated with elevated expression variability, other factors such as copy number variations (CNV) and epigenetics, could also cause variation in gene expression level. To this end, we compiled a list of ∼1, 800 RefSeq genes that reside in CNV regions identified from a recent fine-resolution mapping with pair-end sequencing [50]; however, we did not find the genes showing the highest expression variability are enriched for CNV genes. At the present time, it is difficult to separate the epigenetic effects from genetic effects based on available data, but it is important to note that epigenetic diversity across individuals and among populations can have profound impact in expression variability.

It has long been noted that susceptibility to HIV infection differs greatly among individuals, and individuals infected with HIV also have substantially varied rate of disease progression to full-blown AIDS. To explain such variation in viral resistance, several sequence variants of human genes have been identified, which is best exemplified by CCR5-Δ32 deletion [51], [52] and CCL3L1 copy number variants [39]. By circumventing the identification of the associated sequence variants, our analysis on gene expression posed an important question in understanding the differential HIV susceptibility, i.e. whether examining expression polymorphisms can directly assess such a difference. Our results corroborated such possibility, i.e. host factors interacting with several HIV genes, controlling viral entry, progression and replication cycles, show substantially elevated expression variability among individuals. Interestingly, although host factors involved in immune system are major targets in current HIV research, our results also demonstrated that non-immunity genes that interact with viral genes *nef*, *tat* and *vpr* also show significantly elevated expression variability. This observation might help expand the list of candidate genes that reduce HIV susceptibility. From an evolutionary perspective, our observation might also suggest that the virus can increase the chance of survival by preferentially targeting variable host factors.

## Materials and Methods

### Processing gene expression data

The recently released whole-genome expression profiling of 270 HapMap individuals spanning 4 ethnic populations in the lymphoblastoid cell line [2], [20], includes CHB (Chinese Han in Beijing), YRI (Yoruba people of Ibadan, Nigeria), CEU (U.S. residents with northern and western European ancestry) and JPT (Japanese from Tokyo). Using an Illumina annotation table, we unambiguously mapped 18,127 utilized probes to human mRNA transcripts (only those with RefSeq NM_ identifiers). We then removed the 10% of genes with the lowest expression level (assuming they are not expressed in the lymphoblastoid cell line). The Illumina-annotated gene symbols were mapped onto officially approved HGNC (HUGO Gene Nomenclature Committee) symbols, allowing us to retain a total of 15, 554 unique HGNC genes. We filtered out Y-linked genes, and included both male and female samples in this study since sex-biased expression is minimal (even for X-linked genes) in the lymphoblastoid cell line [20]. We separated expression data of adult children from the unrelated parents because the trio family data might bring unnecessary dependency between data points because of parent-child inheritance in gene expression [2]. Finally we were able to retain 18,081 mRNA transcripts and 15,501 HGNC genes for each of the 30 individuals in both CEU and YRI populations. In addition, we were also concerned with the potential bias caused by the presence of SNPs on the designed microarray probes; however, after mapping the ∼3 million annotated HapMap SNPs onto the 18, 081 Illumina probes, we found the influence is minimal as ∼95% of the probes was not affected.

We used the same expression data as above to identify differentially expressed genes, but the data were median-normalized across composite population by pooling all populations together. This is of vital importance in differential expression analysis because in this way we could normalize the expression profiles of CEU and YRI using the same background scale. By excluding genes showing population-specific variability, we were able to consider 16, 878 transcripts in differential expression analysis.

### HIV-1, human protein interaction

We downloaded the annotated HIV-1, human protein interactions from NCBI (http://www.ncbi.nlm.nih.gov/RefSeq/HIVInteractions/) [27]. We considered human genes having “*all*” interactions with each of the nine HIV-1 genes, and mapped the Entrez ID to RefSeq mRNA IDs by using the DAVID ID conversion tool [26]. After overlapping with the transcripts in our study, we were able to consider 700, 194, 235, 211, 73, 853, 83, 215 and 30 transcripts interacting with HIV-1 genes *env*, *gag*, *nef*, *pol*, *rev*, *tat*, *vif*, *vpr* and *vpu*, respectively.

### Detecting outlier genes by regression analysis for gene expression variability

To identify genes with population-specific expression variability within CEU and YRI, we regressed expression fluctuation, η, in YRI and in CEU reciprocally and derived two lists of genes showing population-specific variation by using CEU and YRI as explanatory variables, respectively. About ∼70% of the genes on one list also appear on another list. The liner model was derived by minimizing the square errors between the observed η and the predicted values (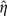). Taking YRI as an example, the residues, 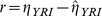, were then normalized and Studentized. For each gene, by fitting a *t*-distribution, we calculated 95% confidence intervals (CIs) of its residue, and the outliers were defined as the genes away from the calculated 95% CIs of the fitted *t*-distribution.

### Extracting annotated promoter, 5′ UTR and 3′ UTRs for human genes

We extracted promoter sequences (annotate by UCSC Genome Browser as upstream 1kb regions from transcription start site), 5′UTR, and 3′UTRs for both outlier genes and all annotated human genes in UCSC.

### Identifying genes showing differential expression

Our protocol is similar as described in [15], in which we performed 10, 000 permutation t-test followed by Benjamini and Hochberg FDR correction.

## Supporting Information

Figure S1 Correlation of expression variability between CEU (parents) and YRI (parents).
(0.13 MB TIF)

Figure S2 Genes that have higher within-population expression variation have higher expected heterozygosity than genome background.
(0.34 MB TIF)

Figure S3 The distribution of expression variability for the differentially expressed genes showing population-specific expression variability.
(0.20 MB TIF)

Figure S4 2D histogram of t-scores between CEU parents and YRI parents, and expression variation in CEU parents (A) and YRI parents (B).
(0.84 MB TIF)

Figure S5 Human genes (CEU and YRI parents) interacting with HIV proteins show elevated expression variability.
(0.29 MB TIF)

Table S1 The complete gene list used in this study, and their expression variability.
(1.56 MB XLS)

Table S2 The list of outlier genes with population-specific variability.
(0.13 MB XLS)

Table S3 The list of genes showing the least and the most variable expression variabilities.
(0.06 MB XLS)

Table S4 The complete list of enriched functional categories for genes showing the least and the most expression fluctuation (only categories with FDR<0.1 are listed).
(0.93 MB XLS)
