# Supplementary figures and images for: Gene Expression Variability within and between Human Populations and Implications toward Disease Susceptibility
Source: PLoS Comput Biol. 2010 Aug 26;6(8):e1000910. doi: 10.1371/journal.pcbi.1000910 (PMC2928754; doi:10.1371/journal.pcbi.1000910)

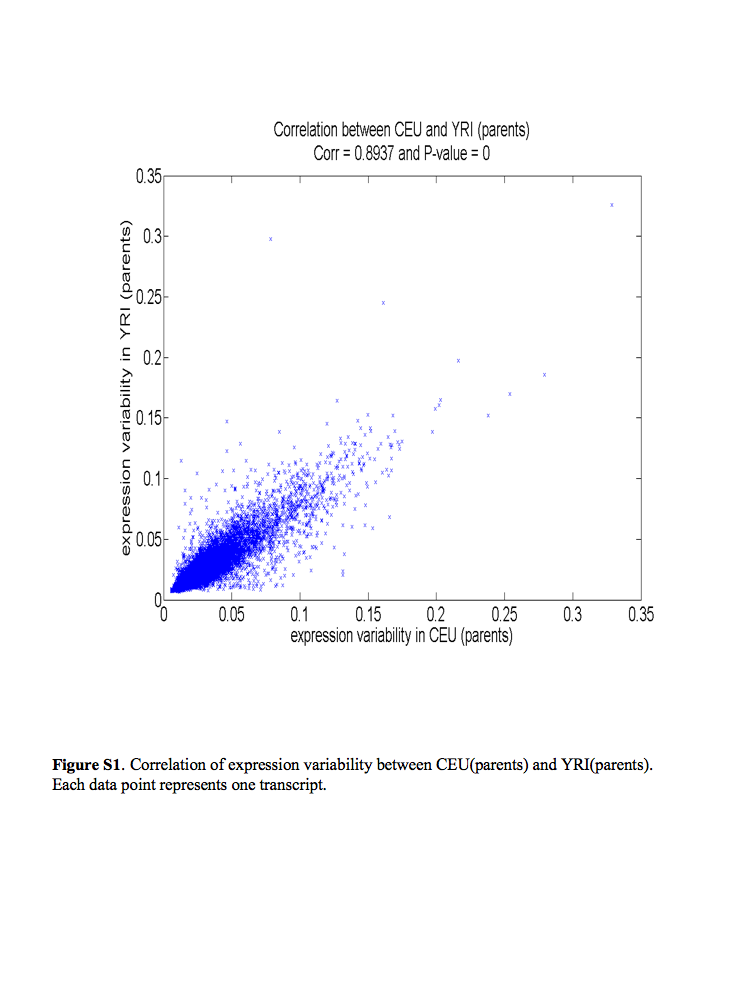

Supplement: Figure S1 — Correlation of expression variability between CEU (parents) and YRI (parents). (0.13 MB TIF) [file pcbi.1000910.s001.tif]

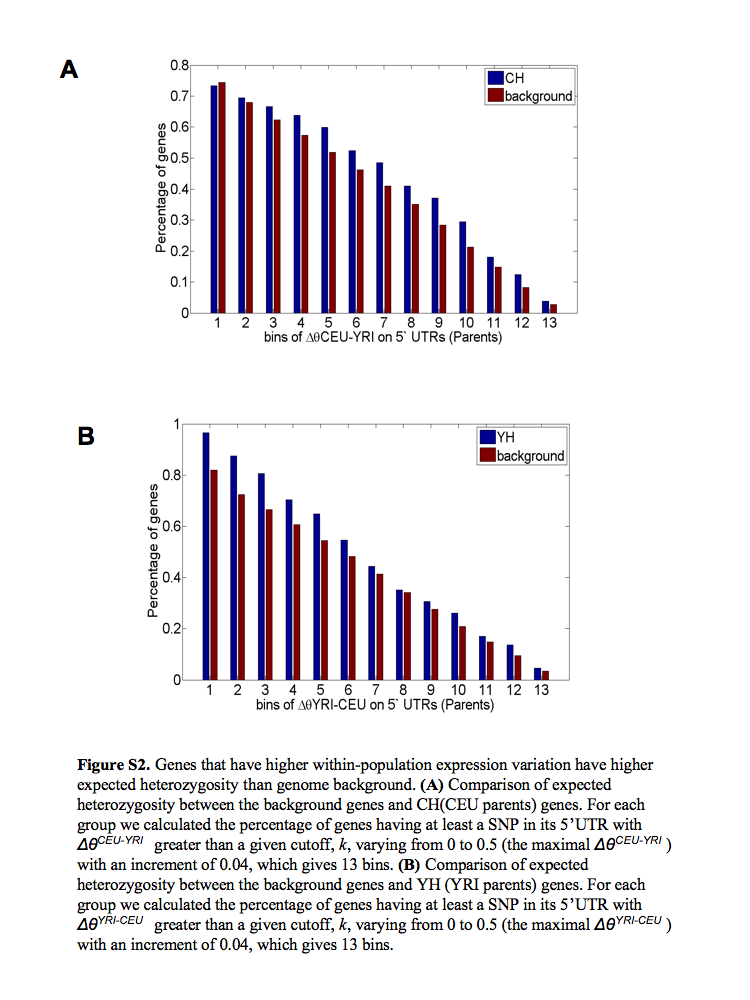

Supplement: Figure S2 — Genes that have higher within-population expression variation have higher expected heterozygosity than genome background. (0.34 MB TIF) [file pcbi.1000910.s002.tif]

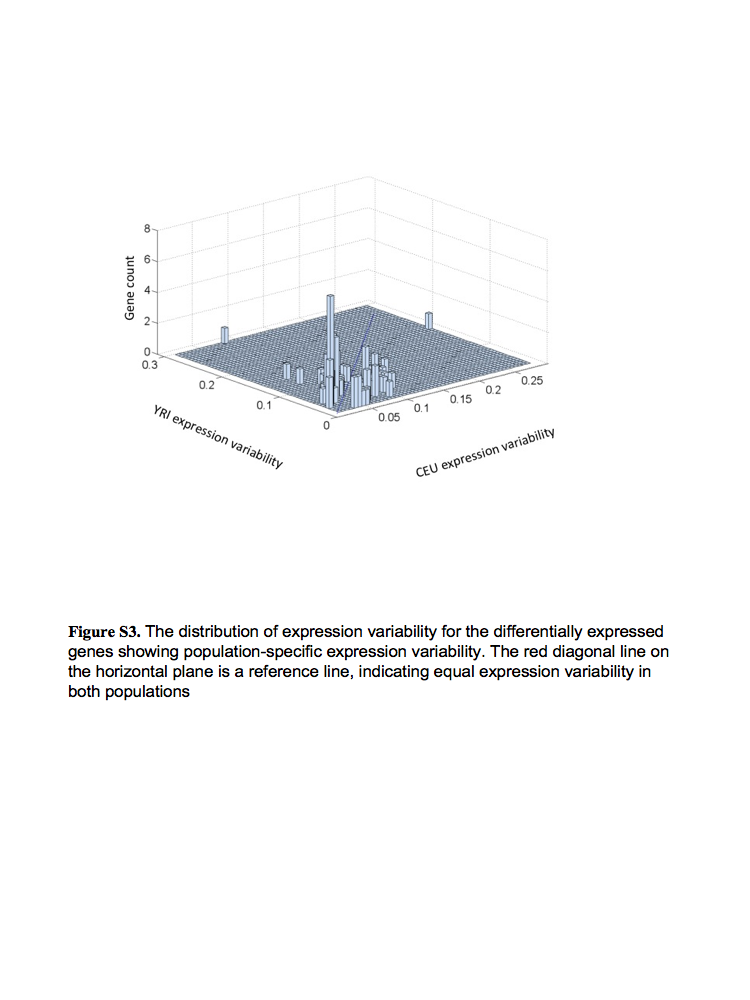

Supplement: Figure S3 — The distribution of expression variability for the differentially expressed genes showing population-specific expression variability. (0.20 MB TIF) [file pcbi.1000910.s003.tif]

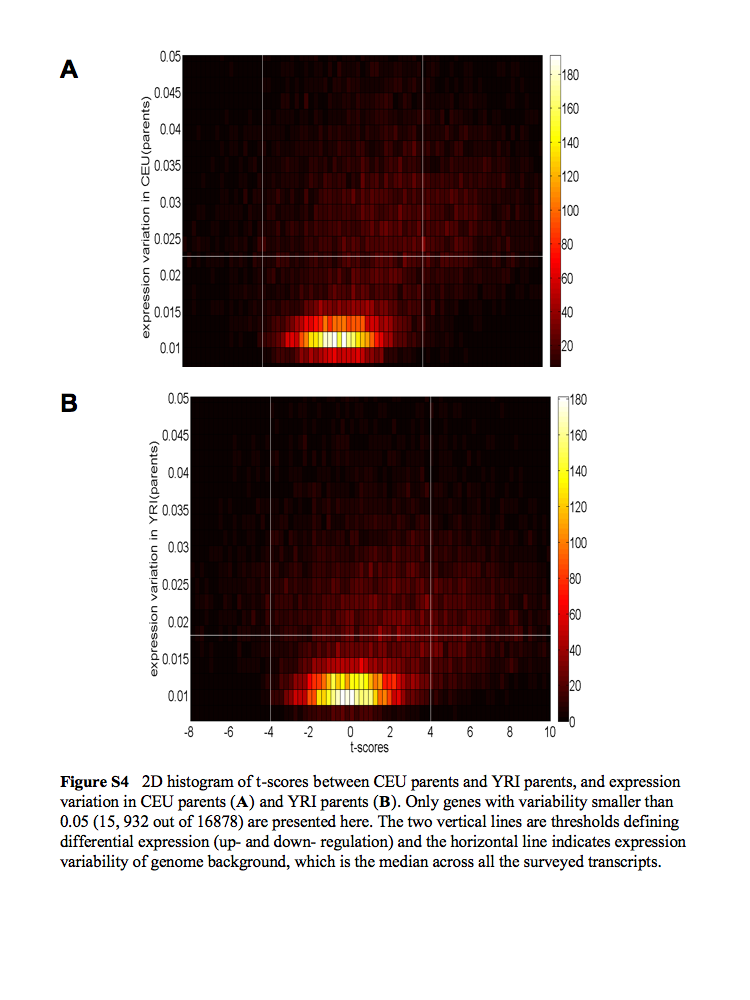

Supplement: Figure S4 — 2D histogram of t-scores between CEU parents and YRI parents, and expression variation in CEU parents (A) and YRI parents (B). (0.84 MB TIF) [file pcbi.1000910.s004.tif]

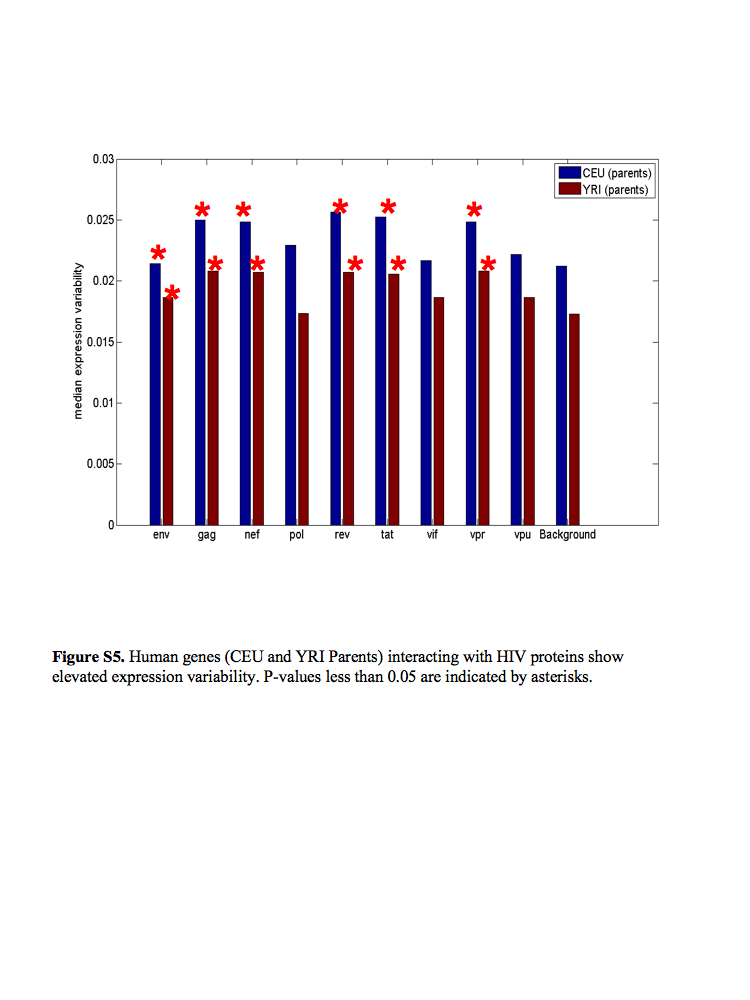

Supplement: Figure S5 — Human genes (CEU and YRI parents) interacting with HIV proteins show elevated expression variability. (0.29 MB TIF) [file pcbi.1000910.s005.tif]
